# Draft genome sequence of extracellular enzyme-producing *Bacillus paralicheniformis* strain NBG-07

**DOI:** 10.1128/mra.00096-24

**Published:** 2024-06-11

**Authors:** Rubab Ramzan, Asad Ali, Muhammad Farooq, Hazrat Ali, Shazia Khaliq, Muhammad Hamid Rashid

**Affiliations:** 1 Industrial Biotechnology Division, National Institute for Biotechnology and Genetic Engineering (NIBGE), Constituent College, Pakistan Institute of Engineering and Applied Sciences (PIEAS), Faisalabad, Pakistan; 2 Agricultural Biotechnology Division, National Institute for Biotechnology and Genetic Engineering (NIBGE), Constituent College, Pakistan Institute of Engineering and Applied Sciences (PIEAS), Faisalabad, Pakistan; The University of Arizona, Tucson, Arizona, USA

**Keywords:** *Bacillus*, enzymes, *paralicheniformis*, whole genome sequence

## Abstract

The genome of *Bacillus paralicheniformis* strain NBG-07 was sequenced using Illumina sequencing due to its ability to produce thermostable enzymes of industrial importance. The strain was isolated from the soil. Annotation of the draft genome revealed genes involved in the production of different enzymes, including alpha-amylase, protease, cellulase, and laccase.

## ANNOUNCEMENT

*Bacillus* is a diverse and large genus, mostly isolated from soil and water. *Bacillus* species are rod-shaped, aerobic, spore-forming, and Gram-positive bacteria, belonging to the family *Bacillaceae* (1, 2). *Bacillus* produce many industrially important thermostable and extracellular enzymes. Demand for thermostable enzymes in various industries, including paper, leather, food, and textile, is increasing day by day (3, 4). *Bacillus paralicheniformis*, in general, produces various thermostable enzymes that can be used in different industrial processes. Genome mining can be performed on the WGS data of this strain to identify potential enzyme candidates involved in the different metabolic pathways.

The strain was collected from the bacterial culture collection of our lab (IE&B Group), Industrial Biotechnology Division, NIBGE; this culture was previously isolated from soil using serial dilution method. Culture from 50% glycerol stock was streaked on LB-agar plates to obtain single isolated colonies. A single isolated colony was inoculated in LB medium (g/L: 10 g tryptone, 10 g NaCl, and 5 g yeast extract) and incubated at 37°C with shaking at 150 rpm. The culture was harvested at equivalent OD_600_ of 8, which contains 4 × 10^9^ bacterial cells. The cells were pelleted down and resuspended in 500 µL of the DNA/RNA shield buffer as directed by MicrobesNG. A volume of 50 µL of this suspension was used for DNA extraction. Following buffer and enzymes were added into the suspension: 120 µL of Tris-EDTA buffer having lysozyme, RNase A, and metapolyzyme (Sigma-Aldrich). The mixture was kept at 37°C for 25 min. Proteinase K (0.1 mg/mL) and 0.5% (vol/vol) SDS were added into the mixture and kept for 5 min at 65°C. After the extraction, DNA was purified, followed by resuspension into 10 mM Tris HCl (elution buffer). An equal volume of solid phase reversal immobilization beads (SPRI) was used to purify DNA from the solution. DNA quantification was done using Quant-iT dsDNA HS (Thermo Fisher Scientific) assay. Nextra XT DNA Library Preparation Kit (Illumina, San Diego, USA) was used for the preparation of DNA Libraries as per protocol. The quantification of DNA and library preparation were done using Hamilton Microlab STAR Automated Liquid Handling System (Hamilton Bonaduz AG, Switzerland).

**Fig 1:**
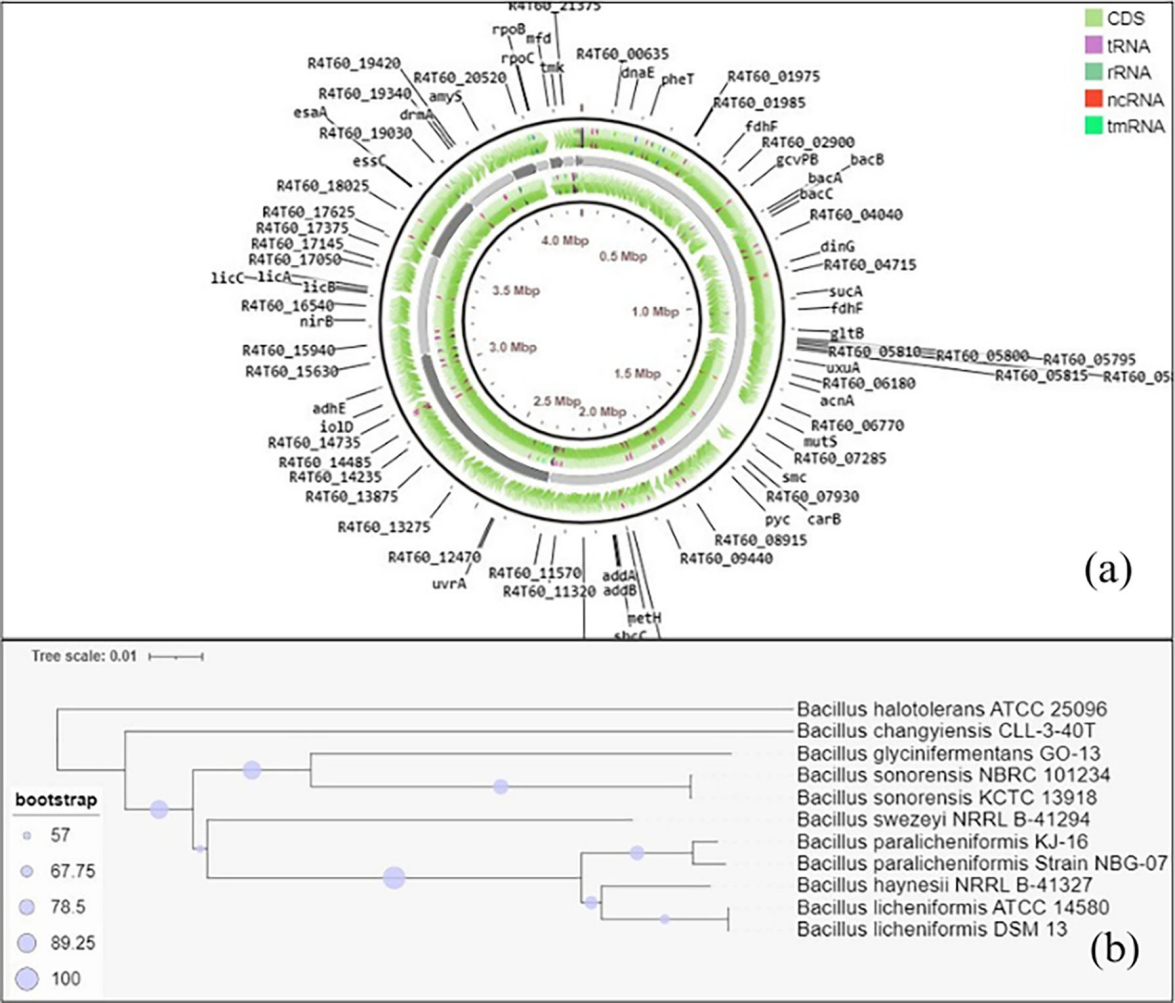
(a) The annotated genome of *Bacillus paralicheniformis* NBG-07. The gray fragments represent the assembled genome consisting of 12 contigs. The inner circle of the CDS is the reverse strand, while the outer circle is the forward strand. Genes located on CDS are labeled outside the circle. (b) Taxonomic inference of NBG-07 strain [A phylogenetic tree inferred using Type Strain Genome Server (TYGS), with 100 bootstraps and utilizing whole genome assembled sequence of the NBG-07 strain.].

Whole-genome sequencing was performed on Illumina NovaSeq6000 (Illumina, San Diego, USA), generating raw paired-end reads of 250 bp. Adaptors were trimmed by using Trimmomatic (version 0.30) (5); a total 479,946 paired-end and 8,752 unpaired reads were retained. The nucleotide sequence quality of the raw reads was assessed using FastQC (version 0.12.1), and reads passing the quality threshold of 30 on the PHRED scale were assembled *de novo* using SPAdes (version 3.15.5) (6) marking genome coverage of ~57×. In total, 12 contigs were obtained with an average *N*_50_ of 2,288,347 bp, corresponding to a 4.2 Mb genome size with a GC content of 45.87% and the largest contig size of 2,288,347 bp. Annotation of the assembled contigs was performed for the identification of different genomic features using NCBI Prokaryotic Genome Annotation Pipeline (7) and visualized using CG View Builder (version 1.1.6) in Proksee (8), as shown in Fig. 1a. The annotation revealed 4,182 protein-coding genes along with 68 tRNA, 15 rRNA, and 5 ncRNA genes (Table 1). Functional annotation of the predicted genes was performed using BlastKoala (version 3.0) (9) and UPIMAPI (version 1.12.3), along with subsystem identification using RAST (version 1.30) (10). Moreover, the phylogenetic tree (Fig. 1b) inferred using TYGS (11) revealed the least evolutionary distance of NBG-07 with *Bacillus paralicheniformis* KJ-16 with an average branch support of 77% and *δ* score of 0.105.

**TABLE 1:** Summary of genome assembly and annotation

Genome	Features
Number of contigs	12
Genome size	4.2 Mb
*L*_50_	1
*N*_50_	2,288,347 bp
Largest contig	2,288,347 bp
GC%	45.87
CDS	4,182
Number of tRNA	68
Number of ncRNA	5
Number of rRNA	15

## Data Availability

This whole-genome shotgun sequencing project has been submitted to GenBank under the accession number JAWRCQ000000000, BioSample accession number SAMN37937522, BioProject number PRJNA1031432, and SRA accession number SRR26846811.
